# Lithium Coordination Complexes and Polymers of 1,4-Diazines

**DOI:** 10.1063/4.0001150

**Published:** 2025-10-27

**Authors:** George D Tisdale, Fahmida Islam, Clifton L Wagner

**Affiliations:** Louisiana State University, Baton Rouge, LA, USA

## Abstract

Lithium-ion batteries have become essential to consumer electronics, electric vehicles, and rechargeable energy storage. However, the common use of cobalt oxides as the cathode material presents ethical and sustainability concerns. One promising class of organic alternatives are 1,4- diazines, with multiple nitrogen-based σ donor sites to coordinate the lithium cation. However, a lack of structural validation of the proposed dual coordination mode helps to limit progress towards the design of competitive organic cathode materials. This motivated the synthesis of lithium coordination complexes with various 1,4-diazines, chiefly among them nitrogen-based polyaromatic heterocycles (nPAHs) quinoxaline and phenazine, ligands that model cathodes through their redox activity and reduction potentials. Use of the hexamethyldisilazane (HMDS) ancillary ligand in nonpolar solvents provided the first structurally characterized lithium azo-arene coordination polymer, alongside other notably neutral species including a metal-organic framework (MOF) and a novel, hydrolyzed morphology of LiHMDS. Extension to the extremely sterically demanding β-diketiminate ligand then provided the projected dual coordination motif. The continued production of lithium coordination polymers motivated reactions of LiHMDS with triethylenediamine and hexamethylenetetramine, producing 1-dimensional chain aggregates that can help furnish the structure-reactivity relationships of these common organolithium reagents. Structural analyses of crystal structures displayed bond lengthening and weaker coordination with higher electron correlation in the nPAHs. The structural results suggest the interactions of lithium ions may involve a significant degree of covalency beyond simple electrostatics that should be considered in further organic electrode design.

